# Tolerance to Excess-Boron Conditions Acquired by Stabilization of a BOR1 Variant with Weak Polarity in Arabidopsis

**DOI:** 10.3389/fcell.2016.00004

**Published:** 2016-02-03

**Authors:** Shinji Wakuta, Teppei Fujikawa, Satoshi Naito, Junpei Takano

**Affiliations:** ^1^Division of Fundamental AgriScience Research, Research Faculty of Agriculture, Hokkaido UniversitySapporo, Japan; ^2^Division of Agrobiology, Graduate School of Agriculture, Hokkaido UniversitySapporo, Japan; ^3^Division of Life Science, Graduate School of Life Science, Hokkaido UniversitySapporo, Japan

**Keywords:** *Arabidopsis thaliana*, Boron, transporter, membrane trafficking, tolerance

## Abstract

Boron (B) is a metalloid that is essential for plant growth but is toxic when present in excess. Arabidopsis BOR1 is a borate exporter, facilitating B translocation from root to shoot under limited-B conditions. BOR1 shows stele side polar localization in the plasma membrane of various root cells, presumably to support B translocation toward the stele. BOR1 is degraded under high-B supply through vacuolar sorting via ubiquitination at the K590 residue to prevent the accumulation of B to a toxic level in shoots. A previous study showed that overexpression of BOR1 under control of the cauliflower mosaic virus 35S RNA promoter improved the growth of Arabidopsis under limited-B conditions without affecting the growth under sufficient-to-excess-B conditions. In this study, we unexpectedly found that ubiquitous expression of a stabilized BOR1 variant improved tolerance to excess-B in Arabidopsis. We established transgenic plants expressing BOR1-GFP fused with hygromycin phosphotransferase (HPT) and BOR1(K590A)-GFP-HPT under control of the ubiquitin 10 promoter. BOR1-GFP-HPT and BOR1(K590A)-GFP-HPT were expressed in various cell types in leaves and roots and showed weak polar localization in root tip cells. BOR1-GFP-HPT, but not BOR1(K590A)-GFP-HPT, was degraded through an endocytic pathway under high-B conditions. Transgenic plants with the stabilized variant BOR1(K590A)-GFP-HPT showed improved root and shoot growth under excess-B conditions. The concentration of B was greater in the shoots of plants with BOR1(K590A)-GFP-HPT or BOR1-GFP-HPT than in those of untransformed wild-type plants. These results suggest that BOR1(K590A)-GFP-HPT confers tolerance to excess-B by excluding B from the cytosol of shoot cells. Results from this study indicate the potential for engineering the trafficking properties of a transporter to produce plants that are tolerant to mineral stress.

## Introduction

Boron (B) is a metalloid that is essential for plant growth and B deficiency is a worldwide problem affecting crop production (Shorrocks, 1997). B is present mainly as boric acid in solution at physiological pH in the absence of interacting molecules. Boric acid is a weak Lewis acid with a p*Ka* of 9.24 [B(OH)_3_ + H_2_O = ${\text{B}(\text{OH})}_{4}^{-}$ + H^+^] (Marschner, 2012). B, as borate,cross-links a pectic polysaccharide, rhamnogalacturonan II, and thus functions in the construction of cell wall structure (O'Neill et al., 2004; Kobayashi et al., 2011). On the other hand, excess-B is toxic to plants. In arid and semi-arid regions, B often accumulates in soil and is toxic to crop plants (Nable et al., 1997). The toxicity probably occurs via binding of borate to *cis*-diol-containing compounds such as ATP, NAD+, and RNA, thereby inhibiting multiple cellular activities (Reid et al., 2004). In root tip cells of Arabidopsis, excess-B was shown to induce DNA double-strand breaks (Sakamoto et al., 2011).

Identification and characterization of boric acid channels and borate transporters revealed adaptive mechanisms of plants under low- and high-B conditions (Takano et al., 2008; Miwa and Fujiwara, 2010; Schnurbusch et al., 2010). In addition to the passive diffusion of boric acid across membranes (Dordas et al., 2000), facilitated transport of B by boric acid channels and borate exporters is required for normal growth under low-B conditions. Arabidopsis NIP5;1 is a boric acid channel localized in the plasma membrane with polarity toward the soil side in the outermost cell layers of roots (Takano et al., 2006, 2010). Arabidopsis BOR1 is a borate exporter localized in the plasma membrane with polarity toward the stele side in various types of root cells (Takano et al., 2002, 2010). The polarized localizations of NIP5;1 and BOR1 appear to be critical factors for the directional transport of B from soil solution to the xylem under low-B conditions. Importantly, the activities of NIP5;1 and BOR1 are down-regulated through post-transcriptional regulation under high-B conditions. NIP5;1 is down-regulated through mRNA degradation that is dependent on the 5′ untranslated region (Tanaka et al., 2011). BOR1 is quickly down-regulated through endocytosis and degradation in vacuoles, dependent on the ubiquitination of the K590 residue under high-B conditions (Takano et al., 2005; Kasai et al., 2011; Yoshinari et al., 2012). These responses are important for preventing the accumulation of B to a toxic level in plant tissues. Polar localization and endocytic degradation have also been reported for other mineral transporters, including the iron transporter AtIRT1, and appear to be common factors controlling mineral transport (Zelazny and Vert, 2014).

In addition to the down-regulation of channels and transporters specifically required for the efficient uptake and translocation of B, B exclusion by borate exporters is an important mechanism for excess-B tolerance. At the single cell level, expression of a BOR1 homolog in yeast *Saccharomyces cerevisiae* lowers cytosolic B concentrations by export at the plasma membrane, thereby conferring excess-B tolerance (Takano et al., 2007). In Arabidopsis, the mRNA levels of *BOR4* were increased two-fold upon excess-B supply, which was dependent on the 5′ untranslated region of *BOR4* (Miwa et al., 2014). BOR4 is localized on the plasma membrane with weak polarity toward the soil side in root cells (Miwa et al., 2007). In T-DNA insertion mutants of *bor4*, the B concentration in shoots was increased and shoot growth was decreased under excess-B conditions, suggesting that BOR4 is involved in B exclusion from roots or restriction of B translocation toward shoots (Miwa et al., 2014). Importantly, BOR4 orthologs in barley and wheat have been shown to function in B exclusion from roots and to be major genetic factors controlling excess-B tolerance among cultivars and landraces (Sutton et al., 2007; Pallotta et al., 2014). A recent phylogenetic analysis suggested that the common ancestor of vascular plants had already acquired BOR1- and BOR4-type transporters for low- and high-B tolerance, respectively (Wakuta et al., 2015).

To date, overexpression of borate transporters has been successfully applied to improve the efficiency of B utilization and excess-B tolerance. Overexpression of BOR1 under control of the cauliflower mosaic virus 35S RNA promoter (35S promoter) improved B translocation to shoots, increased shoot growth, and boosted fertility under low-B conditions in Arabidopsis (Miwa et al., 2006). This approach also improved the growth of tomato plants under low-B conditions (Uraguchi et al., 2014). Enhanced expression of NIP5;1 and the closest BOR1 homolog, BOR2, also improved growth under low-B conditions (Kato et al., 2009; Takada et al., 2014). Furthermore, overexpression of BOR4 decreased the B concentration in roots and shoots and conferred excess-B tolerance in Arabidopsis (Miwa et al., 2007; Miwa and Fujiwara, 2011). Therefore, molecular breeding using B transport proteins is a promising approach to improve crop productivity under variable B conditions.

In this study, we show that ubiquitous expression of a BOR1 variant with restricted polar localization and stable accumulation under high-B conditions confers plant tolerance to excess-B conditions.

## Materials and methods

### Plant material and growth conditions

Col-0 wild-type *Arabidopsis thaliana* (L.) Heynh. was obtained from our laboratory stock. Plants were grown on vertically placed solid media (Takano et al., 2005) in which the boric acid concentrations were adjusted. The solid media contained 1% (w/v) sucrose and 1.5% gellan gum. Surface-sterilized seeds were sown on solid media and incubated for 2 days at 4°C and then at 22°C under a 16-h-light/8-h-dark cycle in a growth chamber. The shoot area was measured on the pictures using the color-range selection tool in photoshop CS5 (Adobe).

### Plasmid construction

Fragments of *proUBQ10, BOR1-GFP, BOR1(K590A)-GFP*, and *HPT*-*NosT* were amplified by PCR using pWaveR131 (Geldner et al., 2009), a plasmid containing BOR1-GFP (Takano et al., 2005), pKKF065 (Kasai et al., 2011), and pGWB505 (Nakagawa et al., 2007), respectively, as templates. The primers used were as follows: for *ProUBQ10*, 5′-atgattacgaattcgagtctagctcaacagagctt-3′ and 5′-aaaagtctcttccatggctgttaatcagaaaaactc-3′; for *BOR1-GFP* and *BOR1(K590A)-GFP*, 5′-atgattacgaattcgcacgacgttgtaaaacgacg-3′ and 5′-ttcaggctttttcatctt gtacagctcgtccatgc-3′; for *HPT-NosT*, 5′-gacgagctgtac aagatgaaaaagcctgaactcac-3′ and 5′-ggccagtgccaagct ctattcctttgccctcgg-3′. The PCR products containing *ProUBQ10, BOR1-GFP* or *BOR1(K590A)-GFP* and *HPT-NosT* were cloned into the *Sac*I and *Hin*dIII sites of pMDC123 using an In-Fusion HD Cloning Kit (Takara), resulting in pSW13 and pSW14, respectively. The inserted DNA fragments were confirmed by sequencing.

### Generation of transgenic plants

To generate transgenic Arabidopsis, the plasmids were used for transformation of Col-0 by the *Agrobacterium*-mediated floral dip method (Clough and Bent, 1998). For selection of transformants, the solid media contained half-strength Murashige-Skoog salt, 1% (w/v) sucrose, 0.8% (w/v) agar, and 7.5 mg l^−1^ bialaphos. T1 transgenic plants were selected by resistance to bialaphos. The bialaphos-resistant T1 plants were transferred onto solid media supplemented with 0.3 μM boric acid and grown for 4–6 days. We then observed GFP fluorescence in roots and shoots under an epifluorescence microscope and selected 11 and 12 plants expressing BOR1-GFP-HPT and BOR1(K590A)-GFP-HPT, respectively, with relatively strong GFP fluorescence intensities. In the T2 progenies of the T1 plants, segregation of hygromycin resistance was examined, and six lines each that were expected to carry a single T-DNA insertion were selected. In the T3 progenies of the T2 plants, segregation of hygromycin resistance was examined, and three lines each were established as homozygous plants. Three lines of each variant (lines 1, 3, and 6 for BOR1-GFP-HPT and 4, 8, and 12 for BOR1(K590A)-GFP-HPT) were used for expression and growth analyses and two lines of each variant (lines 1 and 6 for BOR1-GFP-HPT and 8 and 12 for BOR1(K590A)-GFP-HPT) with good germination rates in the T3 generation were used for determination of B concentrations.

### Imaging analysis

Laser scanning confocal microscopy was performed on a Leica TCS SP8 (Leica Microsystems) equipped with an HCPL APO CS2 x20 water immersion lens, with the following excitation and emission wavelengths: 488 nm and 505–530 nm for GFP, 488 nm and 600–700 nm for FM4-64 (Life Technologies), and 488 nm and 650–750 nm for propidium iodide. FM4-64 was prepared as a 10 mM stock solution in water. Propidium iodide was prepared as a 10 mg ml^−1^ stock solution in water. Plants were transferred from solid to liquid medium containing the dye or inhibitors and incubated at room temperature.

### Quantification of polar localization and B-dependent vacuolar sorting

The polar localizations of BOR1-GFP-HPT and BOR1(K590A)-GFP-HPT were determined by a method described previously (Wakuta et al., 2015). The polarity index is a ratio of fluorescence intensity at the stele side and soil side halves of transverse (apical and basal) plasma membranes in the root epidermis. GFP and FM4-64 fluorescence signals were obtained using laser scanning confocal microscopy (Leica TCS SP8). FM4-64 was used to stain the plasma membrane. The polarity index was calculated as the ratio of GFP fluorescence intensity to that of FM4-64 fluorescence at the stele side divided by that at the soil side. The B-dependent degradation of BOR1-GFP-HPT and BOR1(K590A)-GFP-HPT was examined by quantification of GFP fluorescence. The GFP and FM4-64 fluorescence signals were obtained using laser scanning confocal microscopy (Leica TCS SP8). The intensity of GFP fluorescence in the plasma membrane stained by FM4-64 was measured. Image J (National Institutes of Health, USA) and MetaMorph (Molecular Devices) software were used to quantify the polar localization and B-dependent degradation, respectively.

### Determination of B concentrations in tissues

Plants were grown on solid media. The shoots and roots of plants were harvested and directly transferred into polypropylene tubes. The tissues were dried in an air incubator at 60°C for more than 60 h, and the dry weights were measured. The tissues were digested with 3 ml of 61% nitric acid (for B determination; Wako Pure Chemicals, Osaka, Japan) in a tube at 110°C in a DegiPREP apparatus (SCP Science, Quebec, Canada) until complete dryness. The residues were dissolved in 2% nitric acid and filtered before analysis. ^11^B concentrations in the samples were determined by inductively coupled plasma mass spectrometry (ELAN, DRC-e; Perkin-Elmer). Total B concentrations in the sample were calculated based on natural abundance of B isotopes (^11^B:^10^B = 80.1:19.9). Previous studies indicated that the ratio of B isotopes does not change during the transport in plants (Noguchi et al., 1997, 2000).

### Statistical analysis

Binary comparisons between the wild type and the transgenic lines were performed using Student's *t*-test.

## Results

### Generation of transgenic lines expressing BOR1-GFP-HPT and BOR1(K590A)-GFP-HPT

In our attempt to develop a high-throughput screening assay of mutants defective in BOR1 degradation, we generated transgenic plants expressing BOR1-GFP fused with hygromycin phosphotransferase (HPT) at the C-terminus under control of the ubiquitin 10 (UBQ10) promoter (proUBQ10:BOR1-GFP-HPT). As a control, we also established lines with BOR1(K590A)-GFP-HPT in which the ubiquitination site required for B-dependent vacuolar sorting was substituted with alanine (Kasai et al., 2011). To test whether these lines would be useful for screening based on hygromycin resistance, we tested the growth of lines in solution culture with various concentrations of hygromycin and boric acid.

**Figure 1:**
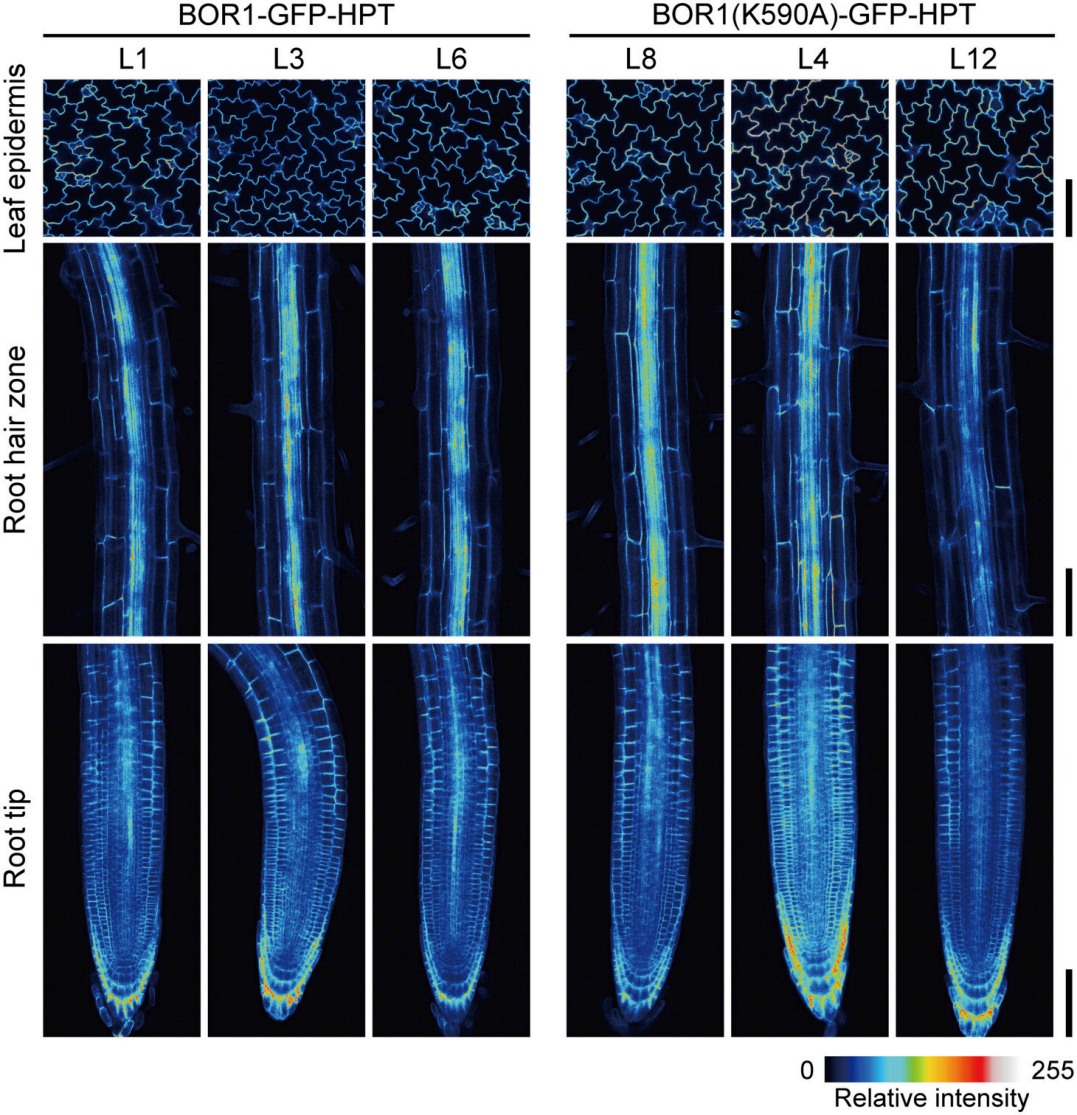
**Comparison of expression levels and patterns of BOR1-GFP-HPT and BOR1(K590A)-GFP-HPT**. Transgenic plants expressing BOR1-GFP-HPT and BOR1(K590A)-GFP-HPT were grown on solid medium containing 0.3 μM boric acid for 5 days. The images were obtained by a laser scanning confocal microscopy at the same settings for the same tissues of independent lines. The intensities of GFP fluorescence in epidermal cells of leaves **(Upper panels)**, the root hair zone **(Middle panels)**, and the root tips **(Lower panels)** are shown as color-coded heat maps. Scale bars represent 100 μm.

Our initial hypothesis was that the stabilized BOR1-GFP-HPT mutant would show hygromycin resistance under high-B conditions. We indeed observed hygromycin resistance of the transgenic lines compared with wild-type plants (Supplemental Figure 1, see difference between wild-type [WT] and BOR1-GFP-HPT at 0 and 20 μg ml^−1^ hygromycin at 5 mM B). However, we unexpectedly found more drastic effects of BOR1(K590A)-GFP-HPT on plant tolerance to excess-B conditions (Supplemental Figure 1). Therefore, we decided to investigate the nature of the high-B tolerance occurring as a result of BOR1(K590A)-GFP-HPT expression. For this purpose, we established three homozygous lines for each of the variants carrying *proUBQ10:BOR1-GFP-HPT* or *proUBQ10:BOR1(K590A)-GFP-HPT*. To compare the pattern and the level of expression among independent transgenic lines, we performed imaging of BOR1-GFP-HPT and BOR1(K590A)-GFP-HPT using laser scanning confocal microscopy. In the transgenic lines, BOR1-GFP-HPT or BOR1(K590A)-GFP-HPT was observed in the epidermal cells of leaves and in the root hair zone and root tips in all cell types (Figure 1). These ubiquitous expression patterns were similar to the previously reported expression patterns of the UBQ10 promoter (Norris et al., 1993; Geldner et al., 2009). The intensities of GFP fluorescence were comparable in each established line (Figure 1).

### Polar localization of BOR1 was affected by the GFP-HPT fusion

**Figure 2:**
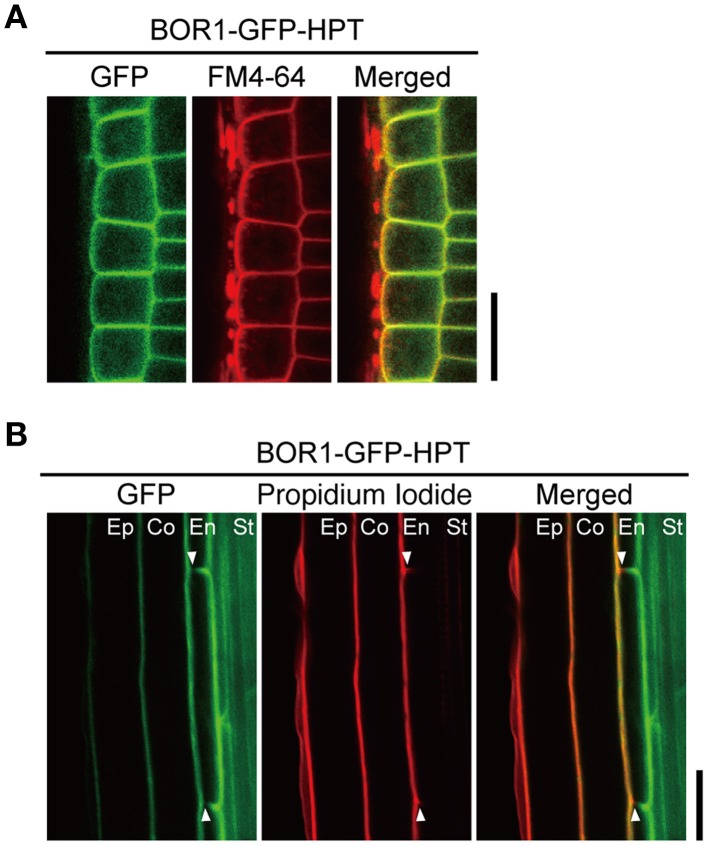
**Polar localization of BOR1-GFP-HPT**. Transgenic plants expressing BOR1-GFP-HPT were grown on solid medium containing 0.3 μM boric acid for 3 days. **(A)** BOR1-GFP-HPT in epidermal cells of the meristem zone. GFP (left), FM4-64 (middle), and a merged image (right) are shown. In the merged images, the GFP (green) and FM4-64 (red) overlapping fluorescence signals appear in yellow. **(B)** BOR1-GFP-HPT in endodermal cells of the differential zone. GFP (left), propidium iodide (middle), and a merged image (right) are shown. Ep, epidermis; Co, cortex; En, endodermis; St, stele. Scale bars represent 25 μm.

We analyzed the intracellular localization of the BOR1-GFP-HPT variants in more detail. Compared to the polar localization of BOR1-GFP toward the stele side (Takano et al., 2010; Wakuta et al., 2015), polarized localizations of BOR1-GFP-HPT and BOR1(K590A)-GFP-HPT were less evident in epidermal cells in the meristem zone (Figure 2A). To confirm the weak polar localization of the variants, the degree of polar localization in the radial direction in roots was quantified by comparing the fluorescence signals of GFP and FM4-64, which stain the plasma membrane in transverse (apical and basal) plasma membrane domains of epidermal cells in the meristem zone (Wakuta et al., 2015). Theoretically, a polarity index >1.0 represents polar localization toward the stele side, while an index < 1.0 represents polar localization toward the soil side. In our previous study investigating the contribution of the evolutionarily conserved trafficking signals of BOR1, the polarity index for BOR1-GFP was 2.0, while those for BOR1(Y373A/Y398A/Y405A)-GFP and BOR1(L455A/L456A)-GFP were 1.2 (Wakuta et al., 2015). These results indicate a weaker polar localization of the BOR1 variants containing mutations in the tyrosine- and dileucine-based trafficking signals. In the cases of BOR1-GFP-HPT and BOR1(K590A)-GFP-HPT, the polarity indexes in each of the two independent lines were ~1.1 [BOR1-GFP-HPT L1, 1.13 ± 0.06; L6, 1.12 ± 0.04; BOR1(K590A)-GFP-HPT L8, 1.07 ± 0.10; L12, 1.12 ± 0.07; mean ± SD, *n* = 60]. These results demonstrate that BOR1-GFP-HPT and BOR1(K590A)-GFP-HPT localize on the plasma membrane with weak polarity in epidermal cells.

We then examined the localization in the endodermis of the root hair zone, where the Casparian strip is developed. The Casparian strip is a diffusion barrier of apoplasts that blocks free diffusion of solutes from the soil into the stele (Geldner, 2013). The Casparian strip also functions as a membrane diffusion barrier to separate two domains of the plasma membrane in the endodermis (Alassimone et al., 2010). In contrast to the weak polar localization in other cell types, BOR1-GFP-HPT was exclusively localized on the plasma membrane of the stele side domain in the mature endodermis (Figure 2B), as was shown for BOR1-GFP (Takano et al., 2010). This was evidenced by the absence of GFP staining in the outer halves of transverse (apical and basal) plasma membranes (Figure 2B, arrowheads). In contrast, propidium iodide, a membrane impermeable dye, stained only the soil side of endodermal cells when applied from outside the roots. Taken together, BOR1-GFP-HPT showed weak stele-side polarity in the root tip cells but clear polarity in mature endodermal cells.

### B-dependent vacuolar sorting was normal in BOR1-GFP-HPT

**Figure 3:**
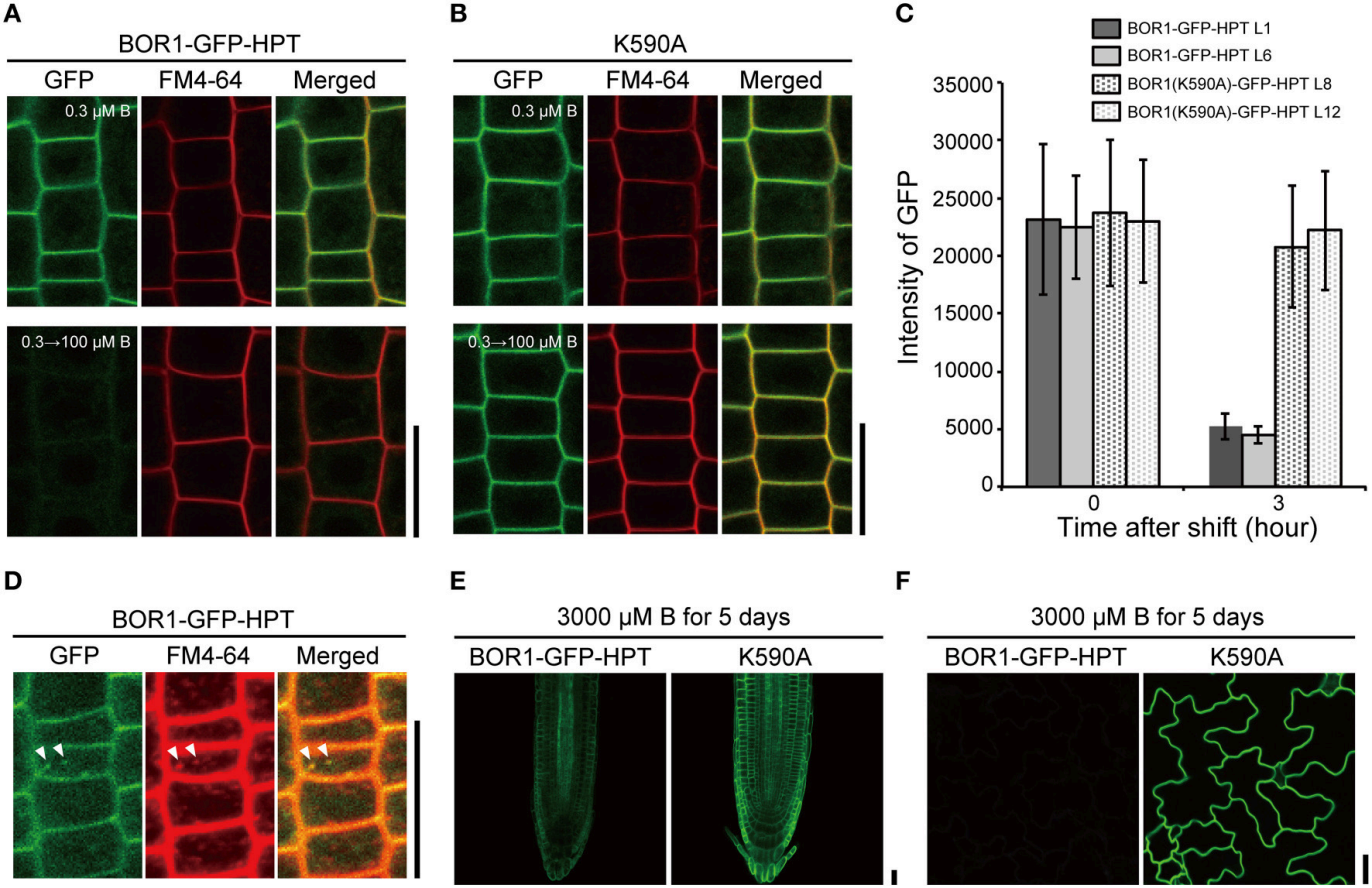
**B-dependent vacuolar sorting of BOR1-GFP-HPT and BOR1(K590A)-GFP-HPT. (A–C)** Transgenic plants expressing BOR1-GFP-HPT and BOR1(K590A)-GFP-HPT were grown on solid medium containing 0.3 μM boric acid for 4 days and then transferred to solid medium containing 100 μM boric acid. BOR1-GFP-HPT **(A)** and BOR1(K590A)-GFP-HPT **(B)** in the root tips grown in 0.3 μM boric acid for 4 days (upper panels), and then shifting to 100 μM boric acid for 3 h (down panels). **(C)** Quantification of fluorescence intensities of BOR1-GFP-HPT and BOR1(K590A)-GFP-HPT in the plasma membrane after shifting to 100 μM boric acid for 3 h. Means ± SD are shown (*n* = 111–149 cells from three roots). **(D)** Co-localization of BOR1-GFP-HPT and FM4-64 after shifting to 100 μM boric acid for 1.5 h. **(E,F)** Transgenic plants expressing BOR1-GFP-HPT and BOR1(K590A)-GFP-HPT were grown on solid medium containing 3000 μM boric acid for 5 days. Root tip **(E)** and leaf epidermal cells **(F)** with BOR1-GFP-HPT and BOR1(K590A)-GFP-HPT. Scale bars represent 25 μm.

We then examined the effect of the HPT fusion on the B-dependent degradation of BOR1-GFP in the endocytic pathway. Transgenic plants were transferred from a low to sufficient boric acid condition (0.3–100 μM) for 3 h and GFP fluorescence was observed in root epidermal cells (Figures 3A,B). BOR1-GFP-HPT disappeared, while BOR1(K590A)-GFP-HPT stably accumulated in the plasma membrane. The GFP fluorescence intensities of BOR1-GFP-HPT in the L1 and L6 lines decreased to 23 and 20%, respectively, whereas those of BOR1(K590A)-GFP-HPT in the L8 and L12 lines remained at 87 and 96%, respectively (Figure 3C). Dotty structures containing BOR1-GFP-HPT were detected after sufficient B supply for 1.5 h (Figure 3D). The dots co-localized with FM4-64, which stains the plasma membrane and membranes in the endocytic pathway (Figure 3D). These results suggest that BOR1-GFP-HPT was internalized into endosomes from the plasma membrane under high-B conditions dependent on K590, similar to the case of BOR1-GFP (Takano et al., 2005, 2010; Kasai et al., 2011). We also grew transgenic plants on a medium containing 3000 μM boric acid for 5 days (Figures 3E,F). BOR1-GFP-HPT was detectable weakly in root tips and scarcely in leaf epidermis under this excess-B condition, while BOR1(K590A)-GFP-HPT was stably accumulated on the plasma membrane in root cells and leaf epidermal cells (Figures 3E,F). Taken together, these data indicate that BOR1-GFP-HPT undergoes normal degradation by B-dependent endocytosis, while BOR1(K590A)-GFP-HPT stably accumulates in various cells under control of the UBQ10 promoter.

### Expression of BOR1(K590A)-GFP-HPT enhanced tolerance under excess-B conditions

**Figure 4:**
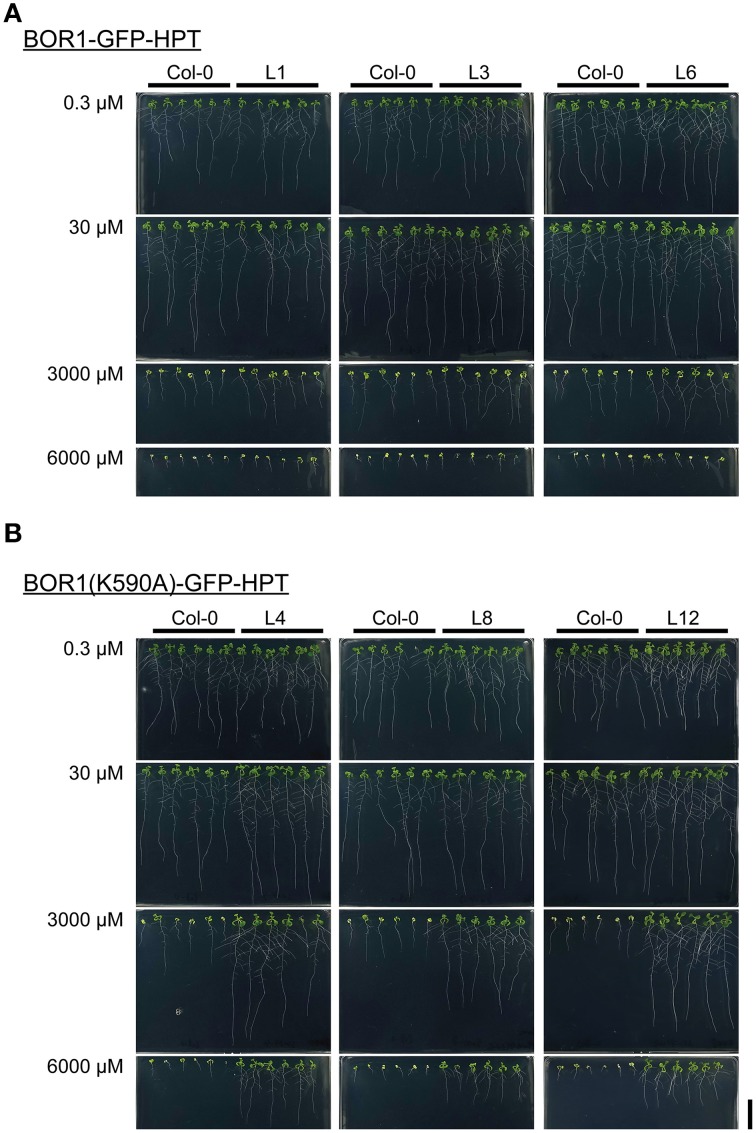
**Growth of transgenic plants expressing BOR1-GFP-HPT (A) and BOR1(K590A)-GFP-HPT (B) under a range of boric acid concentrations**. Wild-type (Col-0) and transgenic lines were grown on solid media containing 0.3, 30, 3000, and 6000 μM boric acid for 9 days. Scale bars represent 20 mm.

**Figure 5:**
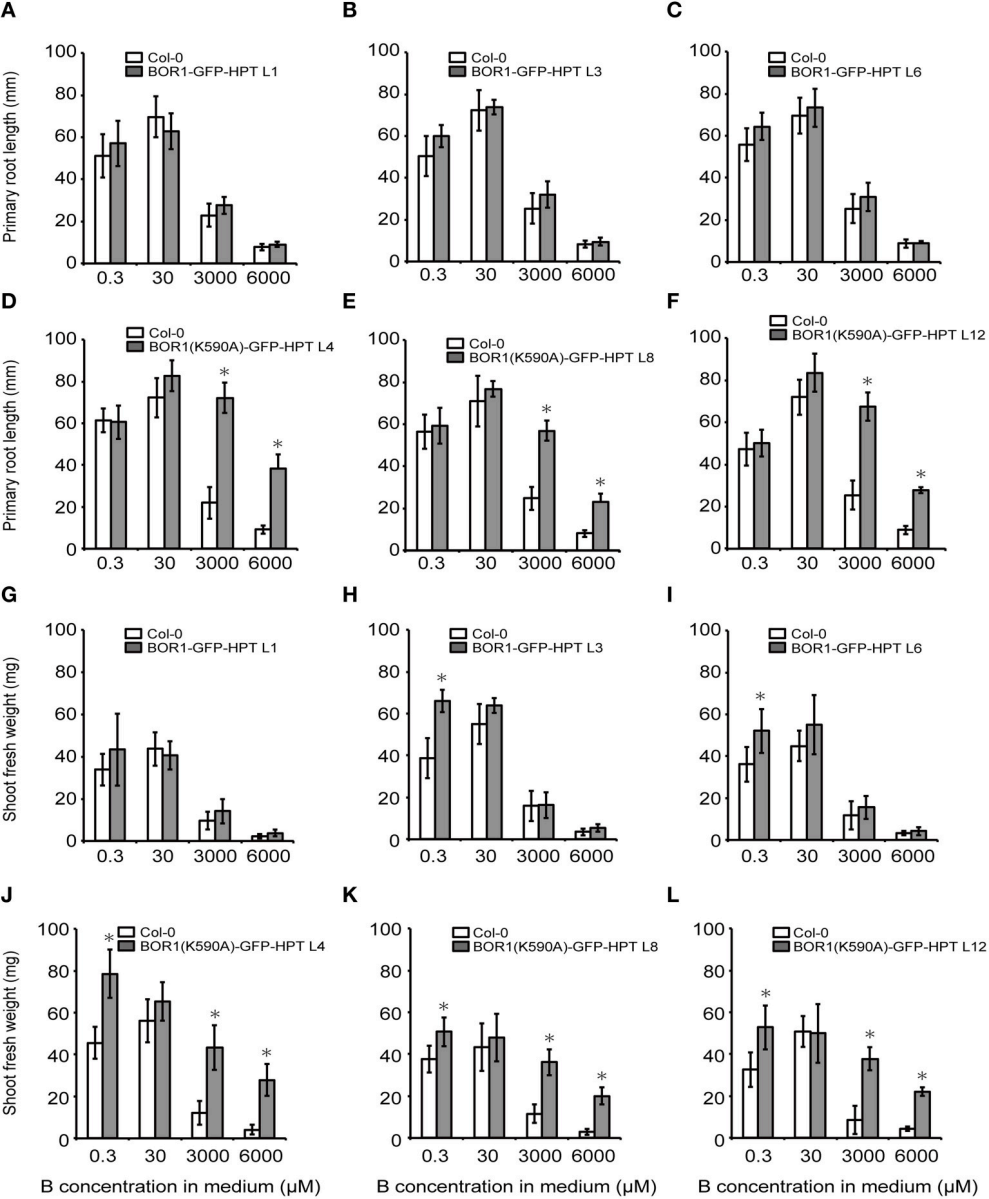
**Root and shoot growths of transgenic plants expressing BOR1-GFP-HPT and BOR1(K590A)-GFP-HPT grown under a range of boric acid concentrations**. The primary root lengths of plants grown for 9 days **(A–F)** and fresh weights of the aerial portion of plants grown for 20 days **(G–L)** were determined. Means ± SD are shown (*n* = 9–12). Asterisks indicate a significant difference compared with wild-type Col-0 under the same conditions by Student's *t*-test (^*^p < 0.01).

To examine the effect of BOR1-GFP-HPT and its stabilized variant on growth, *proUBQ10:BOR1-GFP-HPT* or *proUBQ10:BOR1(K590A)-GFP-HPT* lines were grown on solid media under low- (0.3 μM), sufficient- (30 μM), and excess- (3000 and 6000 μM) B supply. The representative pictures of 9-days old plants are shown in Figure 4. The lengths of main roots (Figures 5A–F) and areas of shoots (Supplemental Figure 2) of 9-days old plants and the fresh weights of shoots of 20-days old plants (Figures 5G–L) were measured. At 0.3 μM B supply, the root growths of all transgenic plants were comparable to those of the untransformed wild-type (Col-0) plants (Figures 4, 5A–F). However, except for the BOR1-GFP-HPT line 1, the fresh weights of the transgenic plants expressing BOR1-GFP-HPT and BOR1(K590A)-GFP-HPT were significantly larger than wild-type plants (*p* < 0.01; Figures 5G–L). These results indicate that ubiquitous expression of the BOR1-GFP-HPT variants contributes to shoot growth under a low-B condition. At 30 μM B supply, the primary root lengths and shoot fresh weights of all transgenic plants were comparable to those of the wild-type (Figures 4, 5). At 3000 and 6000 μM B supply, the primary root lengths and shoot fresh weights of the transgenic plants expressing BOR1-GFP-HPT were comparable to those of wild-type plants (Figures 4A, 5A–C,G–I), while those of the transgenic plants expressing BOR1(K590A)-GFP-HPT were significantly longer and heavier, respectively, than those of wild-type plants (*p* < 0.01; Figures 4B, 5D–F,J–L). The root lengths of BOR1(K590A)-GFP-HPT lines were more than two-fold longer than those of the wild-type plants (Figures 5D–F). The fresh weights of shoots were more than three- and five-fold heavier than those of the wild type at 3000 and 6000 μM B supply, respectively (Figures 5J–L). Consistently, the shoot areas of BOR1(K590A)-GFP-HPT lines were more than two- and four-fold larger at 3000 and 6000 μM B supply, respectively, than those of the wild-type plants (Figures S2D–F). These results indicate that BOR1(K590A)-GFP-HPT greatly improves the tolerance of both root and shoot growth under excess-B conditions.

### Expression of BOR1(K590A)-GFP-HPT did not decrease B concentration in tissues

**Figure 6:**
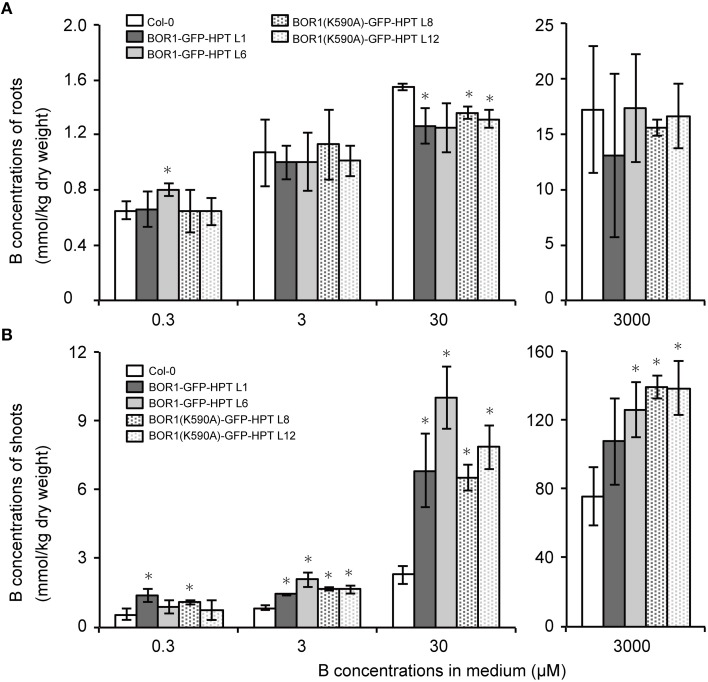
**B concentrations in roots and shoots of transgenic plants expressing BOR1-GFP-HPT and BOR1(K590A)-GFP-HPT**. B concentrations in the roots **(A)** and aerial portion **(B)** of the plants were determined after growth on solid media containing 0.3, 3, 30, and 3000 μM boric acid for 14 days. Means ± SD are shown (*n* = 4). Asterisks indicate a significant difference compared with wild-type Col-0 under the same conditions by Student's *t*-test (^*^p < 0.01).

To investigate the mechanisms underlying the low- and excess-B tolerance of plants expressing BOR1-GFP-HPT variants, we quantified the concentration of B in roots and shoots of plants grown with 0.3, 3, 30, and 3000 μM boric acid. At 3, 30, and 3000 μM B, the concentration of B in the shoots of plants expressing BOR1-GFP-HPT or BOR1(K590A)-GFP-HPT was significantly greater than that in the wild-type Col-0 plants (Figure 6B). However, the concentration of B in the roots was not largely different among the wild-type and transgenic plants (Figure 6A). These results suggest that expression of BOR1-GFP-HPT or BOR1(K590A)-GFP-HPT promotes B translocation toward the shoot under low- to excess-B conditions. Under the excess-B condition (3000 μM B), the order of the shoot B concentration from highest to lowest was as follows: BOR1(K590A)-GFP-HPT lines, BOR1-GFP-HPT lines, and the wild type (Figure 6). This tendency was associated with the accumulation levels of the BOR1-GFP-HPT variant proteins in roots under the excess-B condition (Figure 3E). The higher rate of B translocation in BOR1-GFP-HPT lines than in the wild type was likely due to the detectable accumulation of BOR1-GFP-HPT in the root cells in spite of the down-regulation by endocytosis and degradation (Figure 3E). Taken together, ubiquitous expression of BOR1-GFP-HPT variants resulted in higher B translocation into shoots and also rendered an ability to withstand high-B concentrations. It is likely that the stable accumulation of BOR1(K590A)-GFP-HPT in the plasma membrane prevented B accumulation in the sensitive cytosol by B exclusion to the leaf apoplast, thereby supporting the growth of shoots under excess-B conditions.

## Discussion

Both B deficiency and B excess cause inhibited plant growth. BOR1 exhibits polar localization in the plasma membrane toward the stele side and is required for B translocation toward the xylem under low-B conditions (Takano et al., 2002, 2010). Upon high-B supply, BOR1 is inactivated by B-dependent vacuolar sorting via ubiquitination at the K590 residue to avoid overloading with boric acid (Takano et al., 2005; Kasai et al., 2011). These characteristics of BOR1 are consistent with the result that overexpression of BOR1 under control of the 35S promoter improved tolerance to B deficiency in Arabidopsis by enhancing B translocation without affecting growth under high-B conditions (Miwa et al., 2006). In this study, we found that expression of a BOR1 variant unexpectedly enhanced tolerance to excess B. BOR1(K590A)-GFP-HPT showed restricted polar localization and stable accumulation even under excess-B conditions (Figures 2, 3). The ubiquitous expression of BOR1(K590A)-GFP-HPT by the UBQ10 promoter conferred tolerance to excess-B conditions (Figures 4, 5). This consequence is similar to the case of overexpression of BOR4, a borate transporter stably accumulated under high-B conditions (Miwa et al., 2007; Miwa and Fujiwara, 2011).

However, the mechanism by which B-excess tolerance is conferred appears to be different from those in wild-type Arabidopsis plants and in transgenic plants overexpressing BOR4 under control of the 35S promoter. The concentrations of B in the roots and shoots of plants overexpressing BOR4 were three-fold lower than those in the wild-type plants (Miwa et al., 2007), and the B concentration in shoots of *bor4* loss-of-function mutant plants was greater under high-B conditions (Miwa et al., 2014). These results suggest that BOR4 functions in the exclusion of B from roots to decrease the concentration of B in whole plants under conditions of B-excess. When the plants carrying *proUBQ10:BOR1(K590A)-GFP-HPT* were grown at 3000 μM B supply, the concentration of B in the roots was similar to that in wild-type plants, while the B concentration in the shoots was greater than that of wild-type plants (Figure 6). These results suggest that expression of BOR1(K590A)-GFP-HPT does not exclude B from roots under high-B conditions. Instead, it is likely that BOR1(K590A)-GFP-HPT confers excess-B tolerance by exclusion of B from the cytosol in various cells in shoots. The excess-B tolerance mechanism by B export from the cytosol was also implicated in the leaves of barley and wheat. B-toxicity typically appears as necrosis in leaves in these species. In the leaves of B-tolerant cultivars of barley and wheat, B concentrations that induce necrosis were more than two-fold higher than in those of sensitive cultivars (Reid and Fitzpatrick, 2009). In addition, B concentration in leaf protoplasts from the B-tolerant barley cultivar, Sahara, was only 56% of that from the intolerant cultivar when incubated with 10 mM B. It was also shown that leaching of B from leaves by rain makes significant contribution on growth of roots and shoots of Sahara under excess B conditions. A BOR4 homolog Bot1 was identified as a key factor for excess-B tolerance in Barley. Sahara contains about four times as many *Bot1* gene copies and encodes a Bot1 protein that possesses a higher capacity of B export compared to the intolerant cultivar (Sutton et al., 2007). It is conceivable that *proUBQ10:BOR1(K590A)-GFP-HPT* conferred excess-B tolerance to Arabidopsis plants by a similar B-export mechanism that occurs in the leaves of barley and wheat. In a previous study, overexpression of Arabidopsis TIP5;1, a tonoplast localized aquaporin, under control of the 35S promoter improved tolerance to excess-B conditions in Arabidopsis plants (Pang et al., 2010). Similar to the case with *proUBQ10:BOR1(K590A)-GFP-HPT*, B concentrations in shoots were increased in the TIP5;1 overexpression lines. Although B-transport activity of TIP5;1 has not been proven, the authors proposed that TIP5;1 conferred excess-B tolerance by vacuolar compartmentalization of B. It is likely that protection of sensitive cytosol is a key process in excess-B tolerance of plants.

Another important finding in our study is that the shoot growth of plants expressing BOR1-GFP-HPT and BOR1(K590A)-GFP-HPT tended to be greater than that of the wild-type under low-B conditions (0.3 μM, Figures 4, 5G–L; Supplemental Figure 2). This is attributable to the higher concentration of B in the shoots of the transgenic lines (Figure 6B). Our careful analysis of polar localization showed that BOR1-GFP-HPT and BOR1(K590A)-GFP-HPT maintained weak polarity toward the stele side in root epidermal cells and clear polarity in mature endodermal cells (Figures 2A,B). These results suggest that ubiquitous expression of BOR1 variants enhances B translocation toward the stele side, as was the case of BOR1 overexpression by the 35S promoter (Miwa et al., 2006). The difference in polar localization between BOR1-GFP-HPT variants and BOR4-GFP likely determined the direction of B flux toward the stele in the former case and outside the roots in the latter case.

The results from this study reveal the potential for engineering trafficking properties of transporters to develop stress-tolerant plants. The physiological function of wild-type BOR1 is to effect efficient B translocation under low-B conditions. However, ubiquitous expression of an engineered BOR1 variant with restricted polar localization and stable accumulation conferred excess-B tolerance in plants.

## Author contributions

SW, JT, and SN designed the study and wrote the paper; SW generated transgenic plants and performed growth and imaging analysis; and TF carried out the determination of B concentrations in plant tissues.

## Funding

This work was in part supported by a Grant-in-Aid for Young Scientists (A) (No. 26712007) from the Ministry of Education, Culture, Sports, Science, and Technology of Japan, the NEXT program (GS001) from the Japan Society for the Promotion of Science, and the Young Investigators Grant (RGY0090/2011) from the Human Frontier Science Program, to JT.

### Conflict of interest statement

The authors declare that the research was conducted in the absence of any commercial or financial relationships that could be construed as a potential conflict of interest.

## Abbreviations

35S promoter: cauliflower mosaic virus 35S RNA promoter
B: boron
GFP: green fluorescent protein
HPT: hygromycin phosphotransferase
UBQ10: ubiquitin 10.
