# Supplementary figures and images for: Tolerance to Excess-Boron Conditions Acquired by Stabilization of a BOR1 Variant with Weak Polarity in Arabidopsis
Source: Front Cell Dev Biol. 2016 Feb 3;4:4. doi: 10.3389/fcell.2016.00004 (PMC4737877; doi:10.3389/fcell.2016.00004)

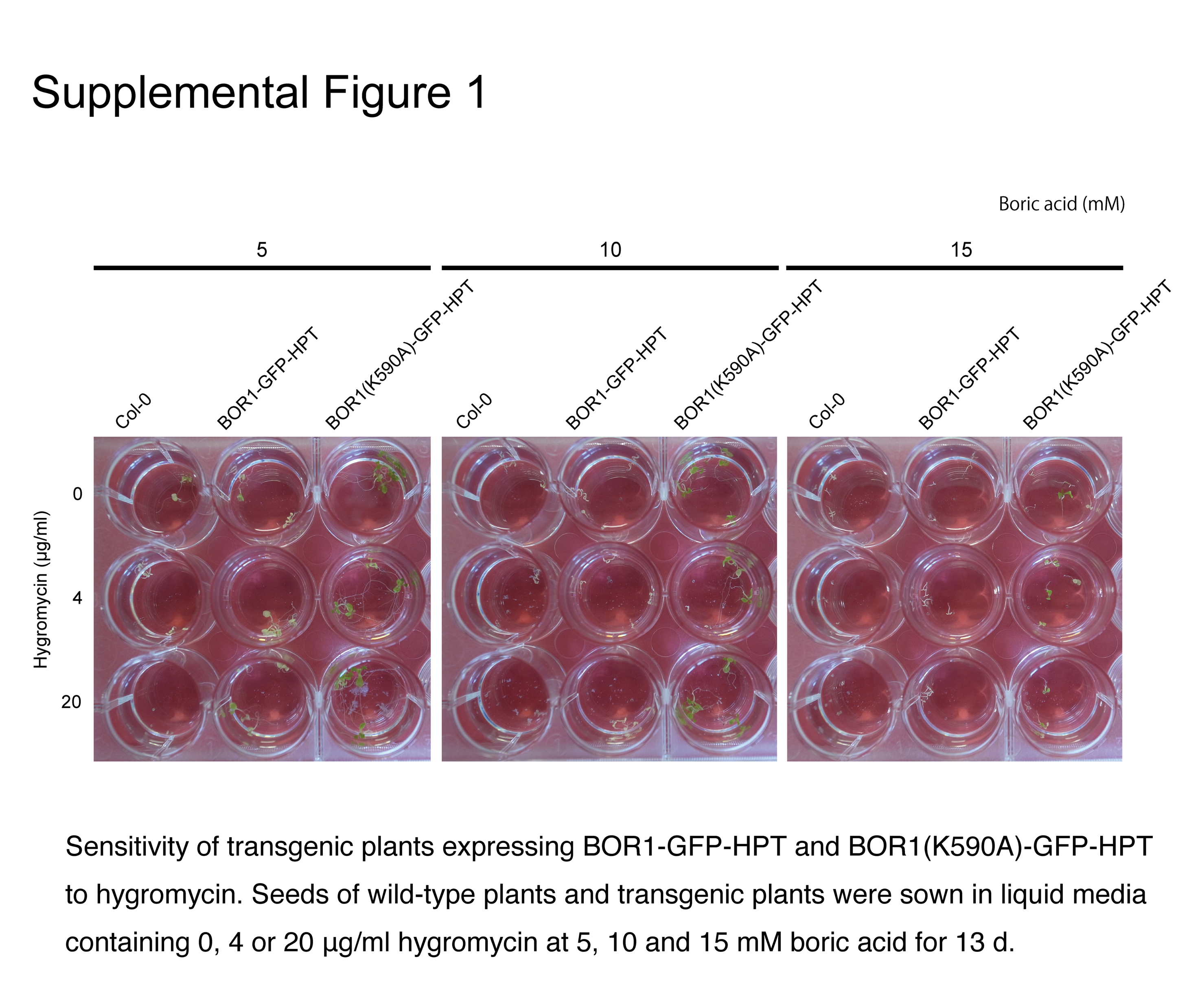

Supplement: Supplementary file 1 [file Image1.TIF]

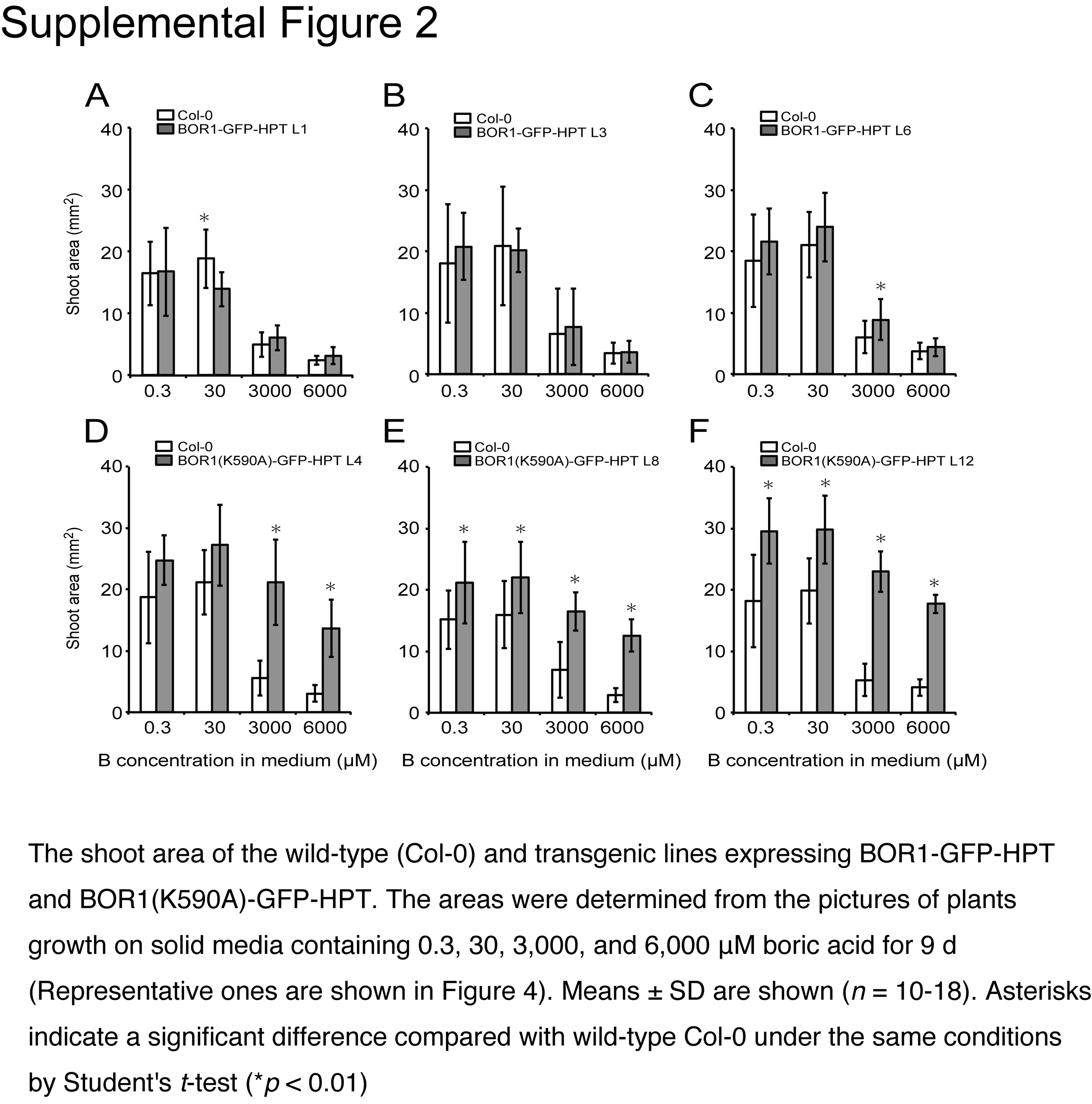

Supplement: Supplementary file 2 [file Image2.TIF]
